# Performance Evaluation of Convolutional Neural Network for Hand Gesture Recognition Using EMG

**DOI:** 10.3390/s20061642

**Published:** 2020-03-15

**Authors:** Ali Raza Asif, Asim Waris, Syed Omer Gilani, Mohsin Jamil, Hassan Ashraf, Muhammad Shafique, Imran Khan Niazi

**Affiliations:** 1 School of Mechanical and Manufacturing Engineering, National University of Sciences and Technology (NUST), Islamabad 44000, Pakistan; araza.bmes18smme@student.nust.edu.pk (A.R.A.); omer@smme.nust.edu.pk (S.O.G.); mohsin@smme.nust.edu.pk (M.J.); hashraf.bmes18smme@student.nust.edu.pk (H.A.); 2 Department of Electrical and Computer Engineering, Memorial University of Newfoundland, St. John’s, P.O. Box 4200, Newfoundland, NL A1C 5S7, Canada; 3 Faculty of Engineering and Applied Sciences, Riphah International University Islamabad, Islamabad 44000, Pakistan; muhammad.shafique@riphah.edu.pk; 4 Center of Chiropractic Research, New Zealand College of Chiropractic, P.O. Box 113-044, Newmarket, Auckland 1149, New Zealand; imran.niazi@nzchiro.co.nz

**Keywords:** classification, deep learning, electromyography, machine learning, myoelectric control, prostheses

## Abstract

Electromyography (EMG) is a measure of electrical activity generated by the contraction of muscles. Non-invasive surface EMG (sEMG)-based pattern recognition methods have shown the potential for upper limb prosthesis control. However, it is still insufficient for natural control. Recent advancements in deep learning have shown tremendous progress in biosignal processing. Multiple architectures have been proposed yielding high accuracies (>95%) for offline analysis, yet the delay caused due to optimization of the system remains a challenge for its real-time application. From this arises a need for optimized deep learning architecture based on fine-tuned hyper-parameters. Although the chance of achieving convergence is random, however, it is important to observe that the performance gain made is significant enough to justify extra computation. In this study, the convolutional neural network (CNN) was implemented to decode hand gestures from the sEMG data recorded from 18 subjects to investigate the effect of hyper-parameters on each hand gesture. Results showed that the learning rate set to either 0.0001 or 0.001 with 80-100 epochs significantly outperformed (p < 0.05) other considerations. In addition, it was observed that regardless of network configuration some motions (close hand, flex hand, extend the hand and fine grip) performed better (83.7% ± 13.5%, 71.2% ± 20.2%, 82.6% ± 13.9% and 74.6% ± 15%, respectively) throughout the course of study. So, a robust and stable myoelectric control can be designed on the basis of the best performing hand motions. With improved recognition and uniform gain in performance, the deep learning-based approach has the potential to be a more robust alternative to traditional machine learning algorithms.

## 1. Introduction

Electromyography (EMG) is a technique to record electrical signals from the muscles during neuromuscular activity [1]. Apart from clinical diagnosis, EMG signals have a wide range of applications in rehabilitation devices [2], electronically controlled chairs [3] and human-computer interactions [4]. For the control of EMG-based upper prosthetic limbs, these signals are used as the main control source for these devices.

Different techniques have been utilized in upper limb artificial devices in order to provide natural and intuitive control for upper limb amputees they are: (1) on–off control, (2) proportional control, (3) direct control, (4) finite state machine control, (5) pattern recognition-based control, (6) posture control schemes and (7) regression control [5,6]. Although these control methods have shown great success, they can only control one device at a time such as a wrist, hand or elbow, so they have limited functionality. The amplitude of the EMG signals or rate of change in EMG is used as a control function in these techniques to change the state of the device. 

Machine learning techniques have evolved over time to provide natural myoelectric control to amputees. These techniques (linear discriminant analysis (LDA), K nearest neighbor (KNN), support vector machine (SVM), artificial neural network (ANN)) assume that each motion of the hand generates a distinct and repeatable set of signals that can be recognized by the pattern recognition (PR) technique. In the literature, some PR techniques are preferred over others based on their signal presentation or feature set. Many studies have focused on optimizing feature sets to bridge the gap between the EMG signals and prosthetic control. Though successful [7], machine learning (ML) algorithms have two major limitations, i.e., feature extraction (features have to be explicitly computed and fed to the network) and the inability to handle large datasets efficiently [8].

The advent of deep learning (an extension of ML) has addressed both these limitations. Deep learning algorithms can intrinsically extract important features for classification and have shown improved efficiency with large datasets [9]. As robustness over time of the classifier is the end goal, deep learning algorithms can be used as an alternative to both conventional and ML algorithms. Most recently deep learning methods have outperformed both conventional methods and ML algorithms [9].

With advancements in deep learning, many studies have explored possibilities for EMG-based hand gesture classification based on the inherent ability of the networks to extract useful features intrinsically. Park and Lee [10] introduced user-adaptive multi-layered CNN for classification of surface EMG (sEMG) from the NinaPro database and concluded that it outperformed SVM by 12%–18%. Atzori et al. [11] showed CNN’s potential compared to traditional techniques (KNN, SVM and LDA). Allard et al. [12] showed CNN’s performance in a real-time application using a wearable sensor for EMG (MYO armband) and achieved 97.8% classification accuracy. [13] CNN’s robustness over time was compared to LDA and auto-encoders SSAE-f (Stacked Sparse Auto-Encoders with features) and SSAE-r (Stacked Sparse Auto-Encoders with raw samples) for data collected over seven days and showed CNN’s improved performance over conventional methods. Studies showed that the classification accuracies of the system changed over time [14]. Zhai [15] proposed a self-calibrating CNN to improve stability and performance over time on the NinaPro dataset and achieved an improvement of 10.18% on DB2 (intact, 50 motions) and 2.99% on DB3 (amputee, 10 motions) compared to an uncalibrated classifier. Chen [16] proposed a “compact” strategy (EMGNet) to reduce the number of parameters involved in the designing of CNN on NinaPro DB5 and achieved slightly better performance compared to classical machine learning algorithms. Huang [17] utilized spectrogram in conjunction with a CNN-LSTM (Long Short-Term Memory network) combination and showed improved classification (from 77.167% to 79.329%) on the NinaPro dataset. Tsinganos [18] proposed a modified CNN and achieved an improvement of 3% on NinaPro dataset. Pinzón-Arenas [19] used CNN to recognize six hand gestures using a wearable EMG recording device (Myo Armband, Thalamic Labs) and achieved a validation accuracy of 98.4% and 99% testing accuracy. 

Normally, the convergence of the network and its variation across the considered range is not discussed, often because more importance is held by the best possible performance (error reduction) of the network. However, a network may converge at multiple points and the chance is random [20]. So, another aspect of this study was to consider performance trends for different sets of hyper-parameters determined by statistically analyzing the performance of each combination (p-values < 0.05 were considered significant). Lastly, a comparison of classification results per learning rate for each individual motion to determine a feasible control strategy (general vs. subject-specific).

## 2. Materials and Methods 

### 2.1. Subjects

The dataset used for the case study was recorded from 18 healthy male subjects (right-handed, aged 20–35 yrs, mean age 26.2 yrs). All participating subjects had no history of neuromuscular disease and congenital upper limb deformities. All subjects volunteered for the experiment and gave written consent before the experiment. a reference electrode (wristband electrode) was placed on the non-dominant hand near the carpus. The data acquisition protocol was approved by the NUST ethical committee Ref# NUST/SMME/BMES/ETH/092019/0042.

### 2.2. Data Acquisition

The surface EMG (sEMG) signals were recorded from the surface with six bipolar Ag/AgCl electrodes from the following muscles: extensor carpi radialis, extensor digitorum muscle, extensor carpi ulnaris, flexor carpi radialis, palmaris longus, flexor digitorum superficialis. Signals were sampled at 8 kHz, bandpass filtered (5–500Hz) and amplified (gain set to 2000 AnEMG12, OT Bioellectronica).

### 2.3. Experiment Protocol

**Figure 1:**
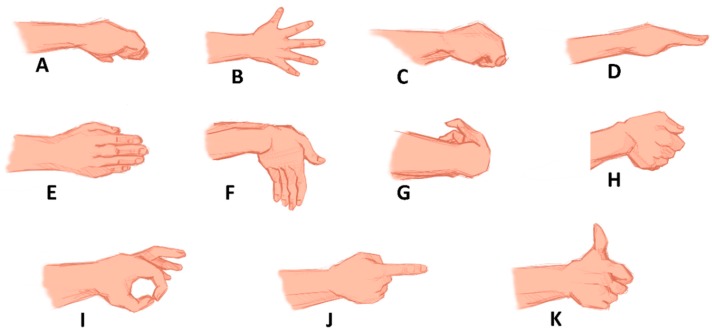
Hand gestures performed by each subject in this study the neutral or rest position are shown in (**A).** The gestures are: (**B**) hand open, (**C**) hand close, (**D**) pronation (forearm), (**E**) supination (forearm), (**F**) extension (wrist), (**G**) flexion (wrist), (**H**) side grip (**I),** fine grip (**J),** pointer and (**K**) agree.

The subjects were asked to perform the following 10 active upper extremity movements (hand open, hand close), flexion (wrist), extension (wrist), pronation (forearm), supination (forearm), side grip (gripping the object perpendicular to the forearm with all fingers flexed and thumb around the object), fine grip, agree and pointer. The motions are displayed in Figure 1. 

Each session comprised of the subject performing four repetitions tasked with 6 s of contraction for each movement, holding the contraction for three to four seconds in each repetition (with medium intensity), as is usually the protocol for data acquisition [21,22]. The contraction time allows sufficient interval for the subject to see the visual cue, initiate contraction, stabilize it and then release to transition into a rest state. The subjects were visually cued for performing the specific movement via the image of the motion through BioPatRec [22], an open-source acquisition Graphical User Interface (GUI) for pattern recognition. The resting time between consecutive contractions was four seconds. In each repetition, the subjects were asked to execute all motions randomly. The acquisition time for each subject was 400 s ([6 s contraction + 4 s rest × 10 motions) and total acquisition time for the complete experiment (18 subjects) was around 3 h.

### 2.4. Convolutional Neural Network (CNN)

Convolutional neural networks are traditional ANNs but with a convolutional layer and dropout layer essentially used to avoid overfitting [23]. Traditional classification and machine learning techniques require feature extraction. These features are decided and computed explicitly and fed into the network. CNN develops several feature detectors on its own, normally known as convolutional layers, and during training sorts the important features required to improve accuracy. This is achieved by convolving the filters with patches of input, creating what is called a receptive field. Receptive fields allow individual filters to incorporate the same weights for learning for all input patches. This field is then fed to the activation function [13]. 

For the network to be able to identify the input more effectively, the classifier needs what we can refer to as “spatial variance”. This ability of the network is gained by pooling. Not only does it help to prevent distortions, but it also reduces the dimensionality of the image reducing the parameters to account for. In order to reduce the problem of vanishing gradient and improve training speed rectified linear units (ReLU) were used as activation function [24]. 

CNN primarily uses images as input the segmented data needs to be “morphed” into a suitable input layer for the network. Since the acquired signal was sampled at 8 kHz and recorded for 40 s, following segmentation, matrices of 6 × 1200 were obtained, where 6 is the number of channels and 1200 is the number samples in each window of 150 ms (8000 Hz × 0.15s).

The architecture of the network comprises of 15 layers. The network has an input layer and three convolution layers with 16, 64, 32 (3 × 3) filters, respectively. The network has three normalization layers and two pooling layers of 2 × 2 and 3 × 3 regions with a stride of 2, respectively. The network also has 3 ReLU layers, a fully connected layer, a SoftMax classification layer and an output layer. 

The training algorithm for the network was stochastic gradient descent with momentum (sgdm). The validation frequency was set to twice in each epoch. The minimum batch size was set to 128 since lower batch values increased the training time. These layers and parameters were chosen empirically.

**Figure 2:**
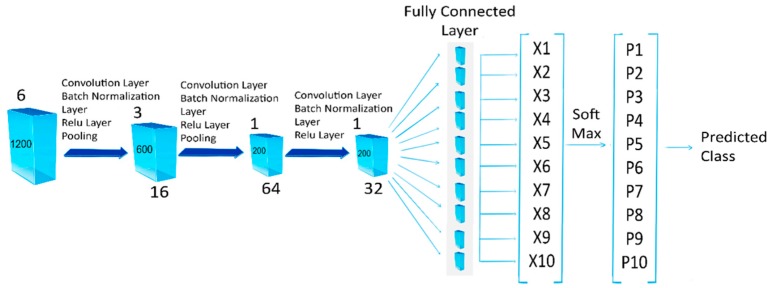
Architecture of the convolutional neural network.

Each motion was labeled as a different class, making a total of 10 classes Figure 2. 

### 2.5. Parametric Optimization

Deep learning neural networks produce exceptional results; training a neural network requires mostly empirical methods to tune hyper-parameters. 

This can never be conclusive since the nature of the signal at hand is completely random and greatly varies from subject to subject; therefore, a generalized set of parameters cannot be obtained. However, the selection of these hyper-parameters greatly affects the obtained results [23]. 

Typically, the deep learning neural network (DLNN) updates by stochastic gradient descent and weights are updated by parameter $\theta^{t}$. Mathematically,

(1) $$\theta^{t}=\theta^{t-1}-\epsilon_{t}\frac{\partial L}{\partial \theta },$$

where L is the loss function and $\epsilon_{t}$ is the learning rate [23].

A smaller learning rate results in the slow convergence of the network inversely, a larger learning rate tends to cause divergence. So, to determine a suitable value, it can take many trials. For example, for the selection of a suitable learning rate, Smith et al. have introduced a Cyclic Learning Rate (CLR) method that tends to reduce the number of iterations required to determine a suitable learning rate. Other methods utilize Bayesian optimization to determine hyper-parameters [24]. 

Another important parameter to be considered is training cycles (Epochs). Tuning of this parameter is perhaps the easiest of all parameters. Given the principle of *early stopping* and provided all the other parameters are set. As during progress, one can observe the number of cycles sufficient for the network to train *Early Stopping* and *Regularization* minimize overfitting with early stopping being the more efficient choice. Although grid search can take several iterations to tune hyper-parameters. It does have one benefit compared to other methods and that it is parallelizable meaning provided enough computing power is available it is possible to instead search for a suitable model that would essentially meet the requirement of the analysis [25]. 

For this specific study, the learning rate was selected to be incremented logarithmically. Since the learning rates are equally spaced for this range. However, in the future, the CLR method can also be employed for the selection of learning rates to improve efficiency. Since the dataset is relatively smaller, a parallel grid search for the suitable learning rate and adequate training cycles is possible and can serve as a decent starting position for getting a general idea of parameter vs. performance and help pave the way later for optimization with different methods for multiple parameters.

The algorithm will grid search through all the possible combinations at different learning rates and different epoch values for each learning rate. Each network is then tested for classification accuracy and the performance metric is the mean classification error (MCE). Lower scores indicate better performance. Obtained results will then be statistically compared and analyzed. 

The experiment was performed on a laptop equipped with a quad-core CPU, 16 GB RAM and 8 GB NVIDIA GTX 1070M GPU. 

### 2.6. Hyper-Parameters

The number of training iterations can be adjusted almost freely since it is one of the more convenient parameters to optimize [25].

The default training iterations in MATLAB are 30. So, a range of 20 to 100 training iterations with increments of 20 is proposed to observe whether or not further training would enhance performance. One hundred epochs were selected as the upper threshold as it does provide sufficient training time and further training yielded negligible performance improvement if any. 

In contrast, if there is only one parameter that deserves attention in terms of optimizing for improving the overall performance of the network, it is the learning rate. The normal value for learning rate ranges from less than 1 to greater than 10^–6^, with 0.01 considered as a standard value for most networks [25]. Hence, the range of 0.00001 to 0.1 was selected.

Scanning between all of these values will require extensive computation and time and the chance of convergence of the network is random, and multiple algorithms have been developed to optimize the learning rate (CLR, Grid Search). The values were incremented logarithmically resulting in a learning rate array of commonly chosen values of [0.00001, 0.0001, 0.001, 0.01, 0.1] in order to observe the general trend of performance of a multi-layered neural network across the range of suggested values. 

### 2.7. Analysis

For performance metrics, the mean classification error for each learning rate and mean classification accuracy for individual subjects were considered. For statistical analysis, a two-way analysis of variance (ANOVA) followed by multiple comparison was implemented to determine performance variation among learning rates. Performance variations were reported significant for p-values less than 0.05.

## 3. Results

### 3.1. Learning Rate vs. Epochs

While we made the argument that sufficient training time is a requirement to train the network appropriately, Table 1 shows that for the learning rates of 0.0001 and 0.001 there is an almost negligible improvement in performance (p-value of 0.58) between training iterations set to 80 and 100 for both learning rates (MCE difference of 0.2% and 0.3%, respectively).

**Figure 3:**
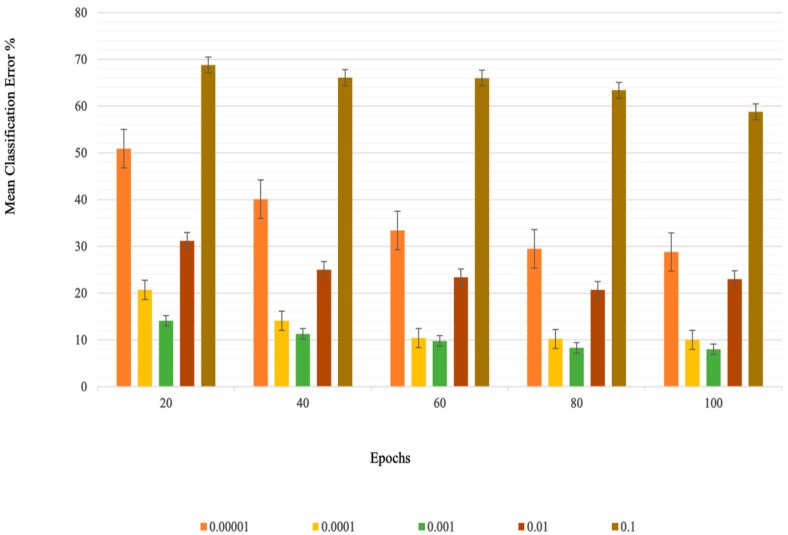
Mean classification error averaged for all subjects for each learning rate across different training iterations (lower is better).

Table 2 shows the overall mean classification error for each learning rate along with standard deviation. Average MCEs for each learning rate at respective epochs are shown in Figure 3. 

### 3.2. Subject-Wise Average Performance

While assessing the performance of individual subjects (Table 2), it can be observed that Subjects 3, 4, 5, 6, 9, 12 and 18 showed improved performance yielding an average classification error of <10% for LR set to 0.001. Multiple factors can be responsible for the other subjects not performing well. The amplitude of the acquired signal can be affected by anatomy, force pattern and muscle fatigue caused during acquisition.

The average classification accuracy for all subjects at each learning rate for different training iterations is given in Table 3.

**Figure 4:**
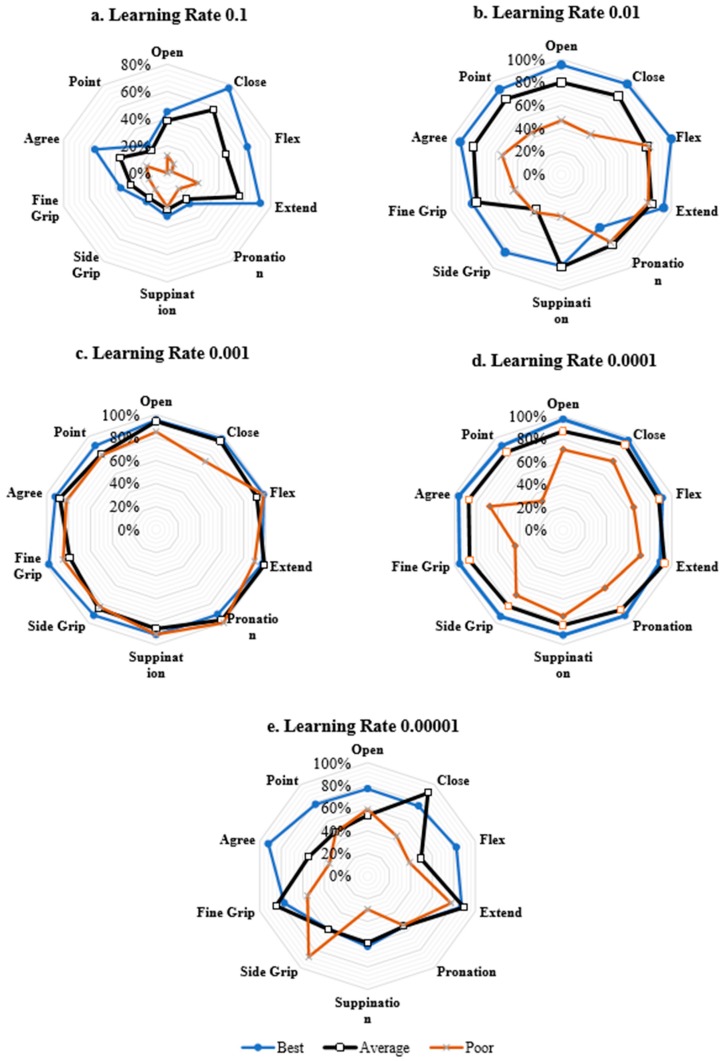
Performance comparison of the network at different learning rates for classification of individual gestures. Values closer to circumference indicate better performance and values closer to origin represent poor performance.

Further comparisons can be drawn between the average performances of the network trained on different learning rates by a visual representation for classification of each class (Figure 4).

When determining the variation in performance between different learning rates, repeated two-way ANOVA, with the two factors being learning rates and training iterations, resulted in the learning rate (LR) 0.0001 significantly outperforming other learning rates (p < 0.05), except 0.001, which significantly outperformed all learning rates (p < 0.05).

From Table 4 we observe that the network on average exhibits better performance when the learning rate is set to either 0.0001 or 0.001 yielding mean classification error ±standard deviation of 13.1% ±4.6% and 10.4% ± 2.5%, respectively, with the network performing the best at LR set to 0.001.

## 4. Discussion

The effect of the selection of considered parameters is crucial to the overall efficiency of the network, a suitable method would be to automate the selection of learning rate by employing a sequential or adaptive technique [23,24,25]. This study is centered around not only the achieved performance of the network but also its effect on each individual motion. It is observable that selecting a suitable learning rate yields a significant change in classification accuracy. Even if the learning rate primarily dictates the overall validation success rate and, in turn, the classification accuracy of the network, it is also essential to determine whether if there is a possibility of obtaining an even smaller value for validation loss (error) beyond the selected value for training cycles. This is why it is necessary to train beyond the set number of epochs to validate this aspect.

During this study, another possible set of parameters was the learning rate set to 0.0001 and epochs set to 100, which gave almost similar results as 0.001 with iterations set to 80 (average difference of 1.4%) obtained p-value of 0.58 in multiple comparison test. 

The trend as shown in Figure 2 clearly depicts that for a smaller learning rate value, the number of epochs should be significantly large for the network to converge [25]. Another interesting validation can be seen when the learning rate is larger, i.e., 0.1. Here we see that the network has completely diverged from learning, resulting in larger values for validation loss and poor training performance. An alternative is the use of random sampling for tuning hyper-parameters, due to the inherent scaling issues of grid search for multiple parameters [25,26,27].

Higher accuracies have been achieved for multiple degrees of freedom through multiple techniques. However, primarily, the need here is to develop patient-specific control strategies. Since it is difficult to obtain a generalized solution for multiple subjects simultaneously (due to the limited number of subjects for testing), the use of adaptive control schemes based on deep learning architectures has become a more viable solution.

Current studies mostly deal with pattern recognition (PR) using different feature sets or proportional control non-invasively using sEMG for improved myoelectric control. However, the increased number of classes results in performance degradation [27]. CNN eliminates the need for traditional feature extraction but at the same time requires multi-layered data as a valid input which requires extra computation for conversion. A suitable substitute method for biosignals would be to implement similarly layered architecture, which utilizes one-dimensional signals as input to reduce computational cost. Since the performance of DLNNs depends on the architecture and optimum parameter selection, an adequate method would either be sequential optimization algorithms [28] or adaptive algorithms to intuitively select optimum parameters.

An extended study could include the selection of multiple hyper-parameters through some sequential or adaptive methods.

For real-time (myoelectric) control, the true limiting variable is time, more specifically the response time of the control system. The response time needs to be adequately small, roughly 300 ms [29], to be unperceivable to the user. As far as time complexity is concerned, it has a 3:1 ratio for training vs. testing time per image due to one forward and two backward propagations [30]. Multiple architectures can have the same time complexity and the run-times are greatly influenced by the GPU’s computing ability. This, in turn, greatly depends on the hardware and application. An attempt [21] at a real-time convolutional neural network discussed its potential. Although the study showed that the CNN potential rivaled that of the standard SVM algorithm, the robustness of CNN over time in comparison to standard pattern recognition methods, and with respect to changes in limb or electrode placement, still needs to be explored. The true limiting factor to make it portable, however, still remains due to the limited processing power available in wearable embedded systems. Currently, wearable embedded systems are not powerful enough to reproduce the same results as a dedicated GPU, but faster learning neural networks with optimized parameters can be used in order to reduce the computational time, which can allow complex models to be implemented on low power devices.

The aim of this study was to observe performance variation of a multi-layered neural network for raw acquired data of several hand motions and observe that whether the network shows similar improvement for all motions or does it prefer some motions over others. The results have shown that the network does tend to perform better when classifying certain motion; however, further investigation is possible by increasing the number of subjects. The aim here is to see the behavior and then in the future make a workaround strategy for improving performance relatively uniformly across all motions.

The study here was constructed around one network architecture and dataset. Further studies may include multiple architectures and performance can be analyzed among various networks across multiple datasets.

## 5. Conclusions

The results exhibit that the network clearly preferred some motions on average across all tested cases. In addition, the network performed similarly at 0.0001 (p > 0.05) against the best guess 0.001 which supports the randomness of convergence. Further analysis can incorporate multiple architectures trained and tested for larger datasets for further validation. 

## Figures and Tables

**Table 1:** Mean classification error for each learning rate vs. epochs.

Learning Rate	Epochs				
	20	40	60	80	100
0.00001	50.9%	40.1%	33.4%	29.5%	28.8%
0.0001	20.7%	14.1%	10.4%	10.2%	10%
0.001	14.1%	11.3%	9.8%	8.3%	8%
0.01	31.2%	25%	23.4%	20.7%	23%
0.1	68.8%	66.1%	66%	63.4%	58.8%

**Table 2:** Subject-wise average performance.

Subjects	Learning Rate Average Classification Accuracy (%)				
	0.00001	0.0001	0.001	0.01	0.1
Subject 1	52.9	63	87.3	86.1	13.3
Subject 2	57.6	89.2	88.4	67.1	31.9
Subject 3	68.8	93.5	92.3	79.3	24.3
Subject 4	70.5	90	92.6	82.3	30.6
Subject 5	74.6	92.6	93.5	86.8	45.5
Subject 6	68.4	90	94.2	75.8	40
Subject 7	57.8	89.6	88.6	67.2	31.6
Subject 8	63.1	85.4	89.4	77.7	39
Subject 9	56	88.3	90.5	73.6	24.4
Subject 10	61	85.4	88.2	67.7	31.5
Subject 11	64	83.3	87.3	77.3	42
Subject 12	66.4	89.4	90	76.6	42.5
Subject 13	61.7	83.3	88.4	81.3	42.3
Subject 14	59	85.8	87.4	53.8	31.6
Subject 15	70.5	90.4	87.5	69.8	32.6
Subject 16	66.8	84.4	86.7	75.1	44.5
Subject 17	63.7	87.9	87.8	73.4	45.9
Subject 18	74.6	92.9	93.1	86.7	45.4

**Table 3:** Average classification accuracy for each learning rate vs. epochs.

Learning Rate	Epochs				
	20	40	60	80	100
0.00001	49.1%	59.9%	66.6%	70.5%	71.2%
0.0001	79.3%	85.9%	89.6%	89.8%	90%
0.001	85.9%	88.7%	90.2%	91.7%	92%
0.01	68.8%	75%	76.6%	79.3%	77%
0.1	31.2%	33.9%	34%	36.6%	41.2%

**Table 4:** Mean classification error (learning rate).

Learning Rate	Mean Classification Error
0.00001	35.7 ±9.2%
0.0001	13.1 ±4.6%
0.001	10.4 ±2.5%
0.01	24.6 ±3.9%
0.1	64.5 ±3.8%

## References

[1] Scheme E., Englehart K. (2011). Electromyogram pattern recognition for control of powered upper-limb prostheses: State of the art and challenges for clinical use. J. Rehabil. Res. Dev..

[2] Phinyomark A., Phukpattaranont P., Limsakul C. (2011). A review of control methods for electric power wheelchairs based on electromyography signals with special emphasis on pattern recognition. Iete Tech. Rev..

[3] Saponas T.S., Tan D.S., Morris D., Balakrishnan R., Turner J., Landay J.A. Enabling always-available input with muscle-computer interfaces. Proceedings of the 22nd Annual ACM Symposium on User Interface Software and Technology.

[4] Yousefi J., Hamilton-Wright A. (2014). Characterizing EMG data using machine-learning tools. Comput. Biol. Med..

[5] Geethanjali P. (2016). Myoelectric control of prosthetic hands: State-of-the-art review. Med Devices.

[6] Roche A.D., Rehbaum H., Farina D., Aszmann O.C. (2014). Prosthetic myoelectric control strategies: A clinical perspective. Curr. Surg. Rep..

[7] Waris A., Niazi I.K., Jamil M., Englehart K., Jensen W., Kamavuako E.N. (2018). Multiday evaluation of techniques for EMG based classification of hand motions. IEEE J. Biomed. Health Inform..

[8] Qiu J., Wu Q., Ding G., Xu Y., Feng S. (2016). A survey of machine learning for big data processing. Eurasip J. Adv. Signal Process..

[9] Schmidhuber J. (2015). Deep learning in neural networks: An overview. Neural Netw..

[10] Park K.-H., Lee S.-W. Movement intention decoding based on deep learning for multiuser myoelectric interfaces. Proceedings of the 2016 4th International Winter Conference on Brain-Computer Interface (BCI).

[11] Atzori M., Cognolato M., Müller H. (2016). Deep learning with convolutional neural networks applied to electromyography data: A resource for the classification of movements for prosthetic hands. Front. Neurorobotics.

[12] Cote-Allard U., Fall C.L., Campeau-Lecours A., Gosselin C., Laviolette F., Gosselin B. Transfer learning for sEMG hand gestures recognition using convolutional neural networks. Proceedings of the 2017 IEEE International Conference on Systems, Man, and Cybernetics (SMC).

[13] Zia ur Rehman M., Waris A., Gilani S., Jochumsen M., Niazi I., Jamil M., Farina D., Kamavuako E. (2018). Multiday EMG-based classification of hand motions with deep learning techniques. Sensors.

[14] Waris A., Niazi I.K., Jamil M., Gilani O., Englehart K., Jensen W., Shafique M., Kamavuako E.N. (2018). The effect of time on EMG classification of hand motions in able-bodied and transradial amputees. J. Electromyogr. Kinesiol..

[15] Zhai X., Jelfs B., Chan R.H., Tin C. (2017). Self-recalibrating surface EMG pattern recognition for neuroprosthesis control based on convolutional neural network. Front. Neurosci..

[16] Chen L., Fu J., Wu Y., Li H., Zheng B. (2020). Hand Gesture Recognition Using Compact CNN Via Surface Electromyography Signals. Sensors.

[17] Huang D., Chen B. Surface EMG Decoding for Hand Gestures Based on Spectrogram and CNN-LSTM. Proceedings of 2019 2nd China Symposium on Cognitive Computing and Hybrid Intelligence (CCHI).

[18] Tsinganos P., Cornelis B., Cornelis J., Jansen B., Skodras A. Deep Learning in EMG-based Gesture Recognition. Proceedings of the PhyCS.

[19] Pinzón-Arenas J.O., Jiménez-Moreno R., Herrera-Benavides J.E. Convolutional Neural Network for Hand Gesture Recognition using 8 different EMG Signals. Proceedings of the 2019 XXII Symposium on Image, Signal Processing and Artificial Vision (STSIVA).

[20] Bergstra J., Bengio Y. (2012). Random search for hyper-parameter optimization. J. Mach. Learn. Res..

[21] Ameri A., Akhaee M.A., Scheme E., Englehart K. (2018). Real-time, simultaneous myoelectric control using a convolutional neural network. PLoS ONE.

[22] Ortiz-Catalan M., Brånemark R., Håkansson B. (2013). BioPatRec: A modular research platform for the control of artificial limbs based on pattern recognition algorithms. Source Code Biol. Med..

[23] Smith L.N. Cyclical learning rates for training neural networks. Proceedings of the 2017 IEEE Winter Conference on Applications of Computer Vision (WACV).

[24] Smith S.L., Le Q.V. (2017). A bayesian perspective on generalization and stochastic gradient descent. arXiv.

[25] Bengio Y. (2012). Practical recommendations for gradient-based training of deep architectures. Neural Networks: Tricks of the Trade.

[26] Glorot X., Bordes A., Bengio Y. Deep sparse rectifier neural networks. Proceedings of the Fourteenth International Conference on Artificial Intelligence and Statistics.

[27] Cho K., Raiko T., Ihler A.T. Enhanced gradient and adaptive learning rate for training restricted Boltzmann machines. Proceedings of the 28th international conference on machine learning (ICML-11).

[28] Bordes A., Bottou L., Gallinari P. (2009). SGD-QN: Careful quasi-Newton stochastic gradient descent. J. Mach. Learn. Res..

[29] Englehart K., Hudgins B. (2003). A robust, real-time control scheme for multifunction myoelectric control. Ieee Trans. Bio-Med Eng..

[30] He K., Sun J. Convolutional neural networks at constrained time cost. Proceedings of the IEEE conference on computer vision and pattern recognition.

